# Global Phylogeography with Mixed-Marker Analysis Reveals Male-Mediated Dispersal in the Endangered Scalloped Hammerhead Shark (*Sphyrna lewini*)

**DOI:** 10.1371/journal.pone.0029986

**Published:** 2012-01-10

**Authors:** Toby S. Daly-Engel, Kanesa D. Seraphin, Kim N. Holland, John P. Coffey, Holly A. Nance, Robert J. Toonen, Brian W. Bowen

**Affiliations:** 1 Department of Zoology, University of Hawaii at Mānoa, Honolulu, Hawaii, United States of America; 2 College of Education, University of Hawaii at Mānoa, Honolulu, Hawaii, United States of America; 3 Hawaii Institute of Marine Biology, University of Hawaii at Mānoa, Kaneohe, Hawaii, United States of America; 4 Harbor Branch Oceanographic Institute, Florida Atlantic University, Fort Pierce, Florida, United States of America; Ecole Normale Supérieure de Lyon, France

## Abstract

**Background:**

The scalloped hammerhead shark, *Sphyrna lewini*, is a large endangered predator with a circumglobal distribution, observed in the open ocean but linked ontogenetically to coastal embayments for parturition and juvenile development. A previous survey of maternal (mtDNA) markers demonstrated strong genetic partitioning overall (global Φ_ST_ = 0.749) and significant population separations across oceans and between discontinuous continental coastlines.

**Methodology/Principal Findings:**

We surveyed the same global range with increased sample coverage (N = 403) and 13 microsatellite loci to assess the male contribution to dispersal and population structure. Biparentally inherited microsatellites reveal low or absent genetic structure across ocean basins and global genetic differentiation (*F*_ST_ = 0.035) over an order of magnitude lower than the corresponding measures for maternal mtDNA lineages (Φ_ST_ = 0.749). Nuclear allelic richness and heterozygosity are high throughout the Indo-Pacific, while genetic structure is low. In contrast, allelic diversity is low while population structure is higher for populations at the ends of the range in the West Atlantic and East Pacific.

**Conclusions/Significance:**

These data are consistent with the proposed Indo-Pacific center of origin for *S. lewini*, and indicate that females are philopatric or adhere to coastal habitats while males facilitate gene flow across oceanic expanses. This study includes the largest sampling effort and the most molecular loci ever used to survey the complete range of a large oceanic predator, and findings emphasize the importance of incorporating mixed-marker analysis into stock assessments of threatened and endangered shark species.

## Introduction

As with other sexually reproducing species, reproductive behavior in sharks has implications for population structure. Females make the greater investment in reproduction with large ova, long gestation times, and time spent transiting to coastal nursery grounds. Males contribute less energy to reproduction and are expected to exhibit promiscuity [1], [2], [3]. The discrepancy between male and female optimal fitness strategies can produce behaviors that influence genetic architecture, including sex-biased dispersal [4]. Previous work in vertebrates has shown mammals to be largely male-biased in dispersal, with females undergoing limited dispersal due to higher site fidelity. This pattern is consistent among highly migratory marine mammals, as recently demonstrated for sperm whales (*Physeter macrocephalus*) in the North Atlantic [5]. In birds, however, the opposite is largely true: male birds establish and defend territories, whereas females move between them, exhibiting female-biased dispersal [6]. Because shark reproduction more closely resembles that of marine mammals than other fishes [7], theoretical expectations are that male-biased dispersal may predominate in this group.

Population structure of coastal-oceanic sharks is something of an enigma, conforming neither to the expectations of sedentary coastal fishes with pelagic larvae, nor the oceanic migrants such as billfish and tunas. Unlike most teleost (bony) fishes, sharks are viviparous, producing small numbers of highly developed young that are capable of swimming and navigation soon after parturition. As a result, many shark species are dependent on shallow coastal habitat for birthing and offspring development [8], [9], [10]. The dichotomy of long-range dispersal ability and coastal reproductive habitat may result in complex population genetic structure, in which female and male (or biparental) markers demonstrate contrasting geographic partitions [11], [12].

Sampling large marine predators is challenging, and few studies have documented phylogeography on a global scale, most of these focusing exclusively on marine mammals (but see [11], [13], [14], [15]). Only three studies have explicitly tested sharks for sex-biased dispersal: the white shark, *Carcharadon carcharias* [16], the shortfin mako shark, *Isurus oxyrhinchus* [14], and the sandbar shark, *Carcharhinus plumbeus* [17], all species that adhere to either coastal or pelagic life histories rather than a combination of the two [18]. In each case, contrasting maternally inherited and biparentally inherited genetic markers indicated dispersive males and philopatric females. The scalloped hammerhead shark, *Sphyrna lewini*, is a large (up to 420 cm) viviparous (live-bearing) shark with a circumglobal distribution in tropical and warm-temperate waters. *S. lewini* gives birth to 13–30 pups following an 8–10 month gestation [19] and is thought to reproduce annually [8], [20]. Pups are born in shallow coastal nursery habitats where they can be seasonally resident for 3–5 years [21]. Adults are highly migratory and have an unusual coastal-pelagic life history, often schooling over seamounts and near continental and insular shelves to depths possibly in excess of 275 m [22]. Adult *S. lewini* can occasionally be found in the open ocean, and documented oceanic movements exceed 1500 km [23]. *S. lewini* populations have declined worldwide as a result of overfishing and bycatch [22], and are listed as globally endangered by the IUCN Red List of Threatened and Endangered Species [24].

An earlier analysis of maternal (mtDNA) markers in *S. lewini* revealed significant genetic structure between oceans and between discontinuous coastlines within oceans [13]. Population structure was low or absent along coastlines and continental margins, a pattern consistent with weak female philopatry to coastal nursery grounds [13], [25], [26]. Duncan *et al.* [13] proposed that females disperse readily across continuous habitat but rarely across open oceans. With limited sampling and a single matrilineal locus, however, the possibility of male-mediated dispersal could not be evaluated.

An ideal system for untangling male and female components of dispersal would include both maternally- and paternally-inherited markers. Lacking markers for the Y sex chromosomes (males carry the XY karyotype; [27]), here we rely instead on biparentally-inherited markers to resolve the male contribution to population structure. Nuclear markers such as microsatellites have been shown to useful for estimating population demographics in marine populations [28], and contrasting nuclear and mitochondrial data have been applied successfully in the past to the identification of differential dispersal patterns between sexes [16], [17], [29], [30]. However few studies to date have addressed a fundamental issue in population genetics that challenges this method, namely the four-fold smaller effective population size (N_e_) of the haploid, uniparental mitochondrial DNA (mtDNA) compared to diploid, biparental nuclear DNA (nDNA) [31], [32], [33]. These differences in N_e_ mean that even in the absence of sex-biased migration, mtDNA structure may be greater than nDNA structure due to the differential rate at which the markers attain drift-migration equilibrium.

**Figure 1:**
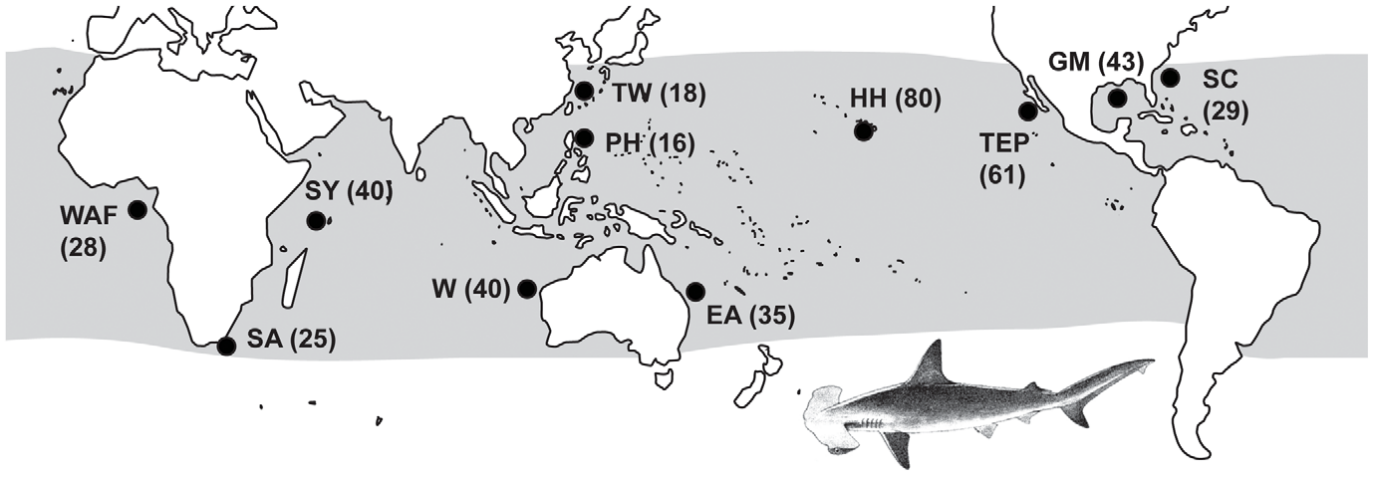
Map showing collection sites. TEP = Tropical East Pacific, HH = Hawaii, TW = Taiwan, PH = Philippines, EA = East Australia, W = West Australia, SY = Seychelles, SA = South Africa, WAF = West Africa, SC = South Carolina, and GM = Gulf of Mexico. Sample numbers for each site are in parentheses.

To illuminate these aspects of *Sphyrna lewini* reproduction and population structure, we used thirteen biparentally inherited microsatellite markers to genotype 403 sharks collected from eleven locations throughout a global range (Figure 1). Our study addresses two primary issues:

- Sex-biased dispersal: Previous mtDNA analyses by Duncan et al (2006) have ruled out high connectivity across ocean basins for female lineages. Nonetheless the circumglobal range and single-species status of *S. lewini* indicates high dispersal. Here we contribute results from 13 microsatellite loci to assess the proposal of sex-biased dispersal in *S. lewini*.
- Population structure and phylogeography: Duncan *et al.* [13] proposed an Indo-Pacific origin for *S. lewini* with subsequent dispersal westward into the Atlantic and eastward into the Central and Eastern Pacific. We address this hypothesis with increased sampling and the application of microsatellite data, which in conjunction with mtDNA data has substantial power to resolve population history.

## Methods

### Tissue collection and ethics statement

Scalloped hammerhead specimens (fin, muscle, or liver tissue) were acquired in Hawaii by fishing under permit #2008-99 issued by the State of Hawaii's Division of Aquatic Resources to the Hawaii Institute of Marine Biology, and elsewhere were bought from commercial fishermen or purchased from fish markets between 1999 and 2008. Specimens were collected from multiple locations in each of three ocean basins, including (i) Pacific: Tropical East Pacific, Pacific Panama, Hawaii, the Philippines, Taiwan, and Eastern Australia; (ii) Indian: Western Australia, Seychelles, and South Africa; and (iii) Atlantic: Western Africa, Gulf of Mexico, and East Coast USA (South Carolina). Collection sites were grouped together if they were within close geographic proximity and statistical analyses indicated no significant differences between sites; two collections of tissue specimens (N = 23 and N = 20, respectively) were made from proximate locations and grouped to create a single Gulf of Mexico collection. Similarly, two sites in Baja California consisting of 32 and 24 specimens were grouped together with five specimens from Pacific Panama to create one Tropical East Pacific (TEP) sample. When possible, specimens were collected from juvenile sharks (fork length <60 cm) within a proposed nursery area to avoid the confounding effect of sampling adults in feeding areas where distinct breeding populations may overlap [12].

### DNA extraction and microsatellite fragment amplification

Tissue samples (<1 cm^3^) were stored in 20% dimethylsulfoxide (DMSO) saturated salt (NaCl) buffer [34] or 75% ethanol (EtOH), and DNA was extracted using a salting-out protocol adapted from Sunnucks and Hales [35]. Individuals were genotyped for thirteen microsatellite loci, including ten species-specific markers [36] and three markers developed for other species (Table 1) [37], [38] following quality control testing as outlined in Selkoe and Toonen [39]. Unlabeled reverse primers were obtained from Integrated DNA Technologies, Inc. (Coralville, Indiana). Forward primers were labeled with 6-FAM, VIC, NED, and PET proprietary dyes (Applied Biosystems, Foster City, California). PCR reactions consisted of 0.1 U Biolase *Taq* DNA polymerase (Bioline; Randolph, Massachusetts), 1× *Taq* buffer, 0.25–0.0625 µM of each primer, 200 µM each dNTP, and 2.0 mM MgCl_2_. PCR amplification on a MyCycler (Bio-Rad; Hercules, California) consisted of an initial denaturation at 95°C for 4 min, followed by 30 cycles of 1 min at 95°C, 30 s at optimal annealing temperature (Table 1), and 30 s at 72°C, followed by a final extension at 72°C for 20 min. PCR products were resolved with an ABI 3100 automated sequencer and visualized using ABI PRISM GeneMapper Software 4.0 (Applied Biosystems).

**Table 1:** Description of the thirteen loci used in this study averaged across all sites.

Locus	*T_a_*	*H_o_*	*H_e_*	*P*	*r*	*A_s_*	*A*
Cli-100^1^	59	0.754	0.813	0.357	−0.048	8.7	16
Pg02^1^	59	0.796	0.804	0.242	−0.004	9.3	20
Sle018	62	0.496	0.629	0.263	0.222	6.5	18
Sle027	62	0.741	0.773	0.328	−0.044	8.5	20
Sle038	62	0.760	0.808	0.343	−0.021	9.5	23
Sle045	62	0.551	0.617	0.467	0.040	4.5	7
Sle053	62	0.765	0.863	0.099	−0.025	13.2	26
Sle054	62	0.515	0.582	0.332	0.121	6.9	19
Sle077	62	0.788	0.919	0.042†	0.133	19.3	52
Sle081	62	0.766	0.823	0.108	0.023	9.3	16
Sle086	62	0.545	0.589	0.387	−0.032	5.6	10
Sle089	62	0.874	0.875	0.550	−0.009	11.5	19
St10^1^	50	0.832	0.882	0.180	−0.004	13.5	25

*T_a_* = annealing temperature, *H_o_* = average observed heterozygosity, *H_e_* = expected average heterozygosity, *P* = significance value from comparison between *H_o_* and *H_e_*, *r* = frequency of null alleles, *A_s_* = average number of alleles per collection site, *A* = total alleles observed at each locus.

1 Non-species-specific markers.

† weakly significant (0.05≥P≥0.01).

We tested for deviation from Hardy–Weinberg Equilibrium (HWE) using Genepop 3.4 [40], and estimated heterozygosity and tested for linkage disequilibrium using Arlequin 3.11 [41]. Genetic duplicates were detected using the Excel Microsatellite Toolkit [42]. Where significant deviations from Hardy–Weinberg Equilibrium were identified, the program MicroChecker 1 [43] was used to determine whether they were consistent with null alleles, errors due to stutter, large allele dropout, scoring errors, or typography. MicroChecker does this by constructing random genotypes from the alleles observed at each locus, and comparing the distribution of these against the distribution of actual observed genotypes across every allele size class. Probabilities for the number of homozygotes within each size class are calculated using a cumulative binomial distribution [44]. MicroChecker was also used to calculate the frequency of null alleles per locus (*r*) according to the algorithm implemented in [43]. Allelic richness for each sampling site was estimated using Fstat 2.9.3.2 [45], which applies a rarefaction method to correct for variation in sample sizes between populations (Table 2). We conducted a regression analysis in jmp 7 (SAS Institute, Cary, NC) to statistically estimate degree of decrease in observed heterozygosity and allelic richness per site with increasing distance from the putative center of origin for *S. lewini*, the Indo-West Pacific, by using straight-line distance from Indonesia in kilometers as our dependent variable.

**Table 2:** Description of the eleven geographic sites sampled for this study, averaged across loci.

Ocean	Site	Abbr.	*N*	*H_o_*	*H_e_*	*A_r_*
Pacific	Tropical East Pacific	TEP	61	0.703	0.754	6.91
	Hawaii	HH	80	0.743	0.783	7.59
	Taiwan	TW	18	0.785	0.791	8.01
	Philippines	PH	16	0.704	0.796	7.65
	East Australia	EA	35	0.763	0.798	8.41
Indian	West Australia	W	28	0.730	0.816	8.54
	Seychelles	SY	40	0.712	0.804	8.49
	South Africa	SA	25	0.745	0.767	7.38
Atlantic	West Africa	WAF	28	0.705	0.784	8.30
	South Carolina	SC	29	0.537	0.586	7.30
	Gulf of Mexico	GM	43	0.703	0.754	4.89

*N* = number of samples per site, *H_o_* = average observed heterozygosity, *H_e_* = expected average heterozygosity, *A_r_* = allelic richness.

### Population genetic analyses

We estimated the degree of genetic differentiation among individual sampling sites with pairwise values of *F*_ST_ generated in Arlequin [41]. Interpreting *F*_ST_ values generated from highly variable multiallelic data can be problematic because the maximum *F*_ST_ is constrained by the mean within-subpopulation heterozygosity [46], [47]. Even in the absence of any shared alleles, highly heterozygous loci will asymptote far below the theoretical maximum (*F*_ST_ = 1), making comparisons between microsatellites and other genetic markers difficult to interpret. To address the downward bias in *F*_ST_ estimates with highly polymorphic loci, we applied the *F*_ST_ standardization approach [46] as implemented by Meirmans [47] in RecodeData 0.1. Pairwise *F*_ST_ estimates differ in magnitude but provide the same qualitative patterns and significance values as standardized *F′*_ST_ estimates (Table 3), so we discuss only the standardized estimates herein.

**Table 3:** Comparison of pairwise *F*_ST_ values between all sampling sites.

	TEP	HH	TW	PH	EA	W	SY	SA	WAF	SC	GM
TEP	0	0.027*	0.114**	0.170**	0.168**	0.152**	0.189**	0.239**	0.172**	0.258**	0.519**
HH	0.006*	0	−0.111	−0.020	−0.059	−0.037	0.072**	−0.018	0.069**	0.080**	0.515**
TW	0.027**	−0.024	0	0.064†	0.035†	−0.002	−0.044	0.027	0.074*	0.092*	0.447**
PH	0.040**	−0.005	0.013†	0	0.077*	0.044	0.037	0.063†	0.085†	0.114*	0.492**
EA	0.038**	−0.012	0.007†	0.016*	0	0.019	−0.003	0.064*	0.103**	0.134**	0.518**
W	0.033**	−0.007	0.000	0.009	0.004	0	−0.010	0.040†	0.057*	0.098*	0.484**
SY	0.040**	0.015**	−0.009	0.007	−0.001	−0.002	0	−0.036	0.086**	0.098*	0.487**
SA	0.059**	−0.004	0.006	0.014†	0.014*	0.008†	−0.007	0	0.071*	0.076*	0.435**
WAF	0.039**	0.015**	0.015*	0.018†	0.021**	0.011*	0.017**	0.016*	0	0.052†	0.313**
SC	0.064**	0.018**	0.021*	0.026*	0.030**	0.021*	0.021*	0.018*	0.012†	0	0.201**
GM	0.175**	0.169**	0.153**	0.170**	0.168**	0.156**	0.157**	0.151**	0.105**	0.070**	0

Observed *F*_ST_ values are shown on the bottom, and *F′*_ST_ is on the top. *F′*_ST_ indicates values that have been corrected using Meirmans' standardization approach for Pairwise *F*_ST_ (Meirmans 2006). TEP = Tropical East Pacific, HH = Hawaii, TW = Taiwan, PH = Philippines, EA = East Australia, W = West Australia, SY = Seychelles, SA = South Africa, WAF = West Africa, SCA = South Carolina, and GM = Gulf of Mexico.

† weakly significant (0.05≥P≥0.01).

*statistically significant (P≤0.01).

**statistically significant (P≤0.001).

The program TreeFit [48] was used to graphically illustrate genetic distances between sampling sites. TreeFit constructs a genetic distance matrix of pairwise *F*_ST_ [49] and then uses these values to build an evolutionary tree where branch lengths reflect genetic distances between sites. The program then generates an R-squared value that statistically expresses the proportion of variation in the distance matrix that is explained by the tree (i.e., how well the figure represents the data). A high R-squared value indicates that the pairwise *F*_ST_s generated in Arlequin are well illustrated by the tree, though it does not provide statistical support for any particular evolutionary model. The evolutionary tree generated in TreeFit was illustrated as a radial dendrogram in TreeView v.1.6.6 [50].

Global patterns of population subdivision were also examined using Structure 2.3.2 [51], [52], which provides an unbiased estimate of the number of gene pools present using a Bayesian model-based clustering method to assign individuals to groups by minimizing linkage disequilbrium and deviations from Hardy-Weinberg expectations. Whereas TreeFit uses pairwise *F*_ST_ to illustrate genetic relationships between sampling sites, Structure's individual-based approach provides a minimum estimate of the number of genetic populations present across *S. lewini's* range. We employed the admixture model with correlated allele frequencies, as this configuration is appropriate in cases of subtle population structure [51], and used collection sites as prior information as per [52]. The admixture model allows individuals in the sample to be assigned to single cluster or jointly to two or more clusters if their combined genotypes indicate admixture. Here, the presence of admixed individuals can provide evidence of unsampled population structure. To infer how many clusters or populations (*K*) are represented in a data set, we plotted the change in the log probability of successive *K* values (Δ*K*) [53] in Jmp 7. Δ*K* cannot be used for *K* = 1, but when more than one population is likely, Δ*K* shows a mode at the actual *K* more consistently than L(*K*) and is particularly effective for resolving large microsatellite data sets where individuals may be admixed [53]. We employed a 10,000 step burn-in followed by 10,000 simulations to test *K* = 1−11 with 10 repetitions each.

Patterns of pairwise differentiation and individual gene pool assignment were used to group collection sites according to the number of populations indicated by Structure. We then assessed the magnitude of genetic differentiation between the *K* subpopulations in Arlequin with a hierarchical Analysis of Molecular Variance (AMOVA; 20,000 permutations), which partitions genetic variation within sites (*F*_ST_), among sites within groups (*F*_SC_), and among groups (*F*_CT_).

### Sex-biased dispersal

We tested for differences between male and female dispersal patterns by comparing pairwise genetic distances between populations for mtDNA and microsatellite DNA (*F′*_ST_). *F*′_ST_ is a standard measure of population differentiation representing the proportion of genetic diversity resulting from allele frequency shifts between sampling sites, with no consideration for genetic distance between individual alleles. For this reason, direct comparisons of *F*′_ST_ with mitochondrial Φ_ST_, which takes into account both haplotype frequency and genetic distance between haplotypes, can be problematic. We therefore used Arlequin to generate a mitochondrial analogue for pairwise *F*′_ST_ (herein identified as mt*F*_ST_) that describes *S. lewini* population structure solely in terms of haplotype frequencies (Table 4). Paired *t* tests of means were performed to examine the difference between mt*F*_ST_, *F*′_ST_ and Φ_ST_ in Jmp 7.

**Table 4:** Pairwise comparisons between key populations for *F*-statistics and migration rates.

Pairwise comparison	*F′*_ST_	mt*F*_ST_	Φ_ST_	M	Nm_1_	Nm_2_
**Along continental margins**						
Taiwan – Philippines	0.064†	0.033	0.100†	2.50	0.49	3.67
Taiwan – E. Australia	0.035†	0.004	0.016	–	–	–
E. Australia – Philippines	0.077*	0.016	0.122†	3.30	0.75	2.02
E. Australia – W. Australia	0.019	0.174**	0.397**	2.20	0.95	0.42
Seychelles – S. Africa	−0.036	0.013	0.009	1.20	0.46	0.20
S. Africa – W. Africa	0.070*	0.604**	0.566**	–	0.06	0.99
Gulf of Mexico – S. Carolina	0.200**	0.400*	0.400*	–	0.26	7.22
TEP – Gulf of Mexico	0.519**	0.656**	0.968**	–	0.32	0.17
Average	0.119	0.237	0.393	2.30	0.47	2.10
**Across ocean basins**						
Hawaii – TEP	0.027*	0.436**	0.448**	0.34	0.85	0.62
Hawaii – Taiwan	−0.111	0.390**	0.330**	0.60	0.31	4.14
Hawaii – E. Australia	−0.059	0.209**	0.171**	1.20	1.10	2.32
S. Africa – W. Australia	0.039†	0.450**	0.991**	0.06	0.34	0.44
Seychelles – W. Australia	−0.009	0.521**	0.736**	0.02	0.69	0.31
S. Africa – S. Carolina	0.076*	0.534**	0.573**	0.08	–	–
W. Africa – S. Carolina	0.051†	0.540**	0.817**	–	–	–
W. Africa – Gulf of Mexico	0.312**	0.911**	0.972**	–	1.52	0.03
Average	0.041	0.499	0.629	0.38	0.80	1.31

Pairwise nuclear *F′*_ST_ values are from microsatellite data, with mt*F*_ST_ and Φ_ST_ values [13] for mitochondrial DNA. M = number of migrants per generation for mitochondrial markers alone [data from 13]. Nm_1_ = estimated migrants per generation into population 1 (on the left) from population 2 (on the right) for mixed mitochondrial and nuclear markers. Nm_2_ = estimated migrants per generation into population 2 from population 1 for mixed mitochondrial and nuclear markers.

† weakly significant (0.05≥P≥0.01).

*statistically significant (P≤0.01).

**statistically significant (P≤0.001).

We estimated migration rates (*m_1_* and *m_2_*; Table 4) using the coalescent-based program IMa [54] on the CBSU computing clusters at Cornell University. IMa simulates gene genealogies using MCMC sampling methods, and implements an “isolation with migration” model that does not assume gene flow and genetic drift are in equilibrium. Although IMa2 [55] can handle multiple populations at once, we analyzed all adjacent pairs of populations separately in IMa because IMa2 requires a well-supported phylogeny of the groups of individuals being analyzed [55]. Although some runs were performed on nuclear data alone, MCMC mixing and convergence were poor, so these results are not reported and mtDNA and nDNA data sets were combined for all IMa analyses. We started with an analysis in “MCMC Mode” using the full complement of model parameters (i.e., θ_1_≠θ_2_≠θ_A_, and *m_1_*≠*m_2_*), with broad priors for all, and reduced them in repeated runs to better sample the posterior distribution. Once several replicates converged on the same answer, we recorded the maximum likelihood estimate (MLE) for each parameter and 95% highest posterior density interval or HPD. We converted migration parameters *m_1_* and *m_2_* into the number of migrants per generation (Nm) using the equation Nm = (θ *m*)/4.

## Results

Microsatellite fragments were analyzed for 403 specimens from eleven sampling locations. Micro-Checker [43] detected no errors resulting from DNA degradation, low DNA concentrations, and primer-site mutations. Microsatellite Toolkit for Excel found no evidence of duplicate or redundant sampling among individuals. Null alleles were detected at low frequencies (*r*; Table 1) among populations at three loci: Sle018, 054, and 077. These nulls were generally too rare to affect HWE, although Sle077 had a significant *P* value (*P* = 0.042) consistent with departure from equilibrium (Table 1). To test whether marginal (though non-significant) differences between expected vs. observed heterozygosities at some loci could confound population level analyses, we removed the four loci that showed that largest difference between H_o_ and H_e_ (Cli-100, Sle018, Sle053, and Sle077) and re-ran pairwise *F*_ST_. A comparison of pairwise *F*_ST_ values calculated from the subset of 9 loci and from the full data set was non-significant (paired t-test calculated in jmp; t = 1.99, df = 64, *P* = 0.325). As such, we present results from the full 13 loci here. There was no evidence of linkage disequilibrium among pairs of loci after correction for multiple comparisons.

Observed heterozygosity among geographic sampling sites ranged from *H_o_* = 0.537−0.785, and allelic richness ranged from *A_r_* = 6.91−8.54 with the exception of the Gulf of Mexico (*A_r_* = 4.89; Table 2). Observed heterozygosities were highest in Taiwan, East Australia, and South Africa, while allelic richness was highest in East Australia, West Australia, and the Seychelles (*A_r_* = 7.30−8.49). The East Pacific and the Gulf of Mexico sites had the lowest heterozygosity and allelic richness (*A_r_* = 4.89−6.91). Allelic richness showed significant decrease with increasing distance from Indonesia (R-square = 0.43, *P* = 0.028), while the decrease of observed heterozygosity was lower and not statistically significant (R-square = 0.25, *P* = 0.113).

**Figure 2:**
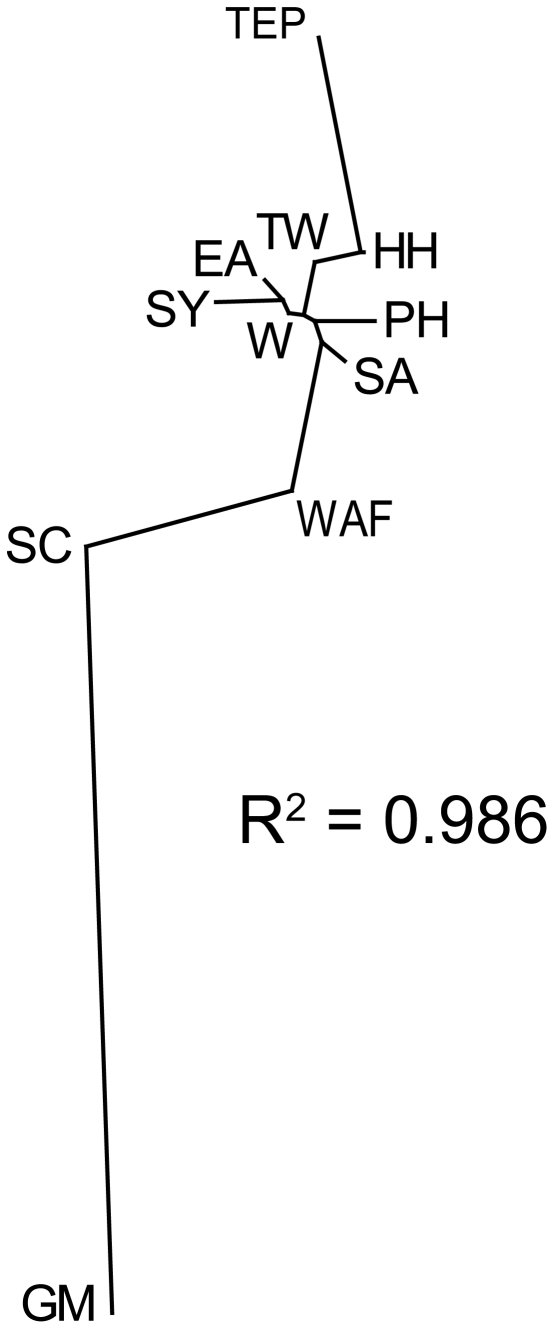
Unrooted radial dendrogram of pairwise *F*_ST_ genetic distances generated from microsatellite genotypes using TreeFit [**48**]. Lengths of braches demonstrate allelic similarities between nodes, each of which represents a separate sampling site. R-square value expresses the proportion of variation in the distance matrix that is explained by the dendrogram. TEP = Tropical East Pacific, HH = Hawaii, TW = Taiwan, PH = Philippines, EA = East Australia, W = West Australia, SY = Seychelles, SA = South Africa, WAF = West Africa, SC = South Carolina, and GM = Gulf of Mexico.

Both corrected (*F′*_ST_) and uncorrected (*F*_ST_) microsatellite based estimates of pairwise population differentiation revealed a general pattern of high contemporary gene flow across ocean basins and along continental margins (average *F′*_ST_≈0), with some noteworthy exceptions (Table 3). Significant levels of genetic structure were detected across the 2000 km of open ocean separating Hawaii from the Tropical East Pacific (TEP; *F′*_ST_ = 0.027, *P* = 0.001), however no comparisons differentiated Hawaii from the three sampling sites in the West Pacific (average *F′*_ST_ = −0.063, *P* = 0.874). No population structure was detected across the Indian Ocean (e.g. between West Australia and the Seychelles, *F′*_ST_ = −0.010, *P* = 0.670). Likewise Indian Ocean samples are undifferentiated from Taiwan, Philippines, and Eastern Australia in the West Pacific (average *F′*_ST_ = −0.018, *P* = 0.470), indicating contemporary gene flow bridging the Sunda Shelf barrier. South Africa was weakly differentiated from most other Indo-Pacific populations except for the Seychelles, with which it shares a continuous continental shelf (*F′*_ST_ = −0.036, *P* = 0.956). While the degree of differentiation indicates some isolation of the South African site, this site shows allele frequencies similar to Hawaii across a distance of 17,600 km (*F′*_ST_ = −0.018, *P* = 0.764). Between the Indian and Atlantic Oceans, South Africa is differentiated from West Africa (*F′*_ST_ = 0.071, *P* = 0.006). Low but significant structure also differentiates West Africa and South Carolina (*F′*_ST_ = 0.052, *P* = 0.042), indicating limited contemporary gene flow across the Atlantic. The Gulf of Mexico was highly differentiated from every other site sampled (average *F′*_ST_ = 0.438, *P*<0.001), including the proximate South Carolina site in the West Atlantic (*F′*_ST_ = 0.201, *P*<0.001). The highest level of allelic differentiation separates the Gulf of Mexico from the TEP (*F′*_ST_ = 0.519, *P*<0.001). The broad mixing of microsatellite alleles across the Indian and Pacific oceans is clearly illustrated in the radial dendrogram of pairwise *F*_ST_ genetic distances (Figure 2), which connects all Indo-Pacific sampling sites with the exception of the TEP (Hawaii, Taiwan, Philippines, East Australia, West Australia, Seychelles, and South Africa) together with short intervening branch lengths. TreeFit assigned an R-squared value of 0.986, indicating that this figure describes the genetic distance reflected in the data set with a high degree of confidence.

**Figure 3:**
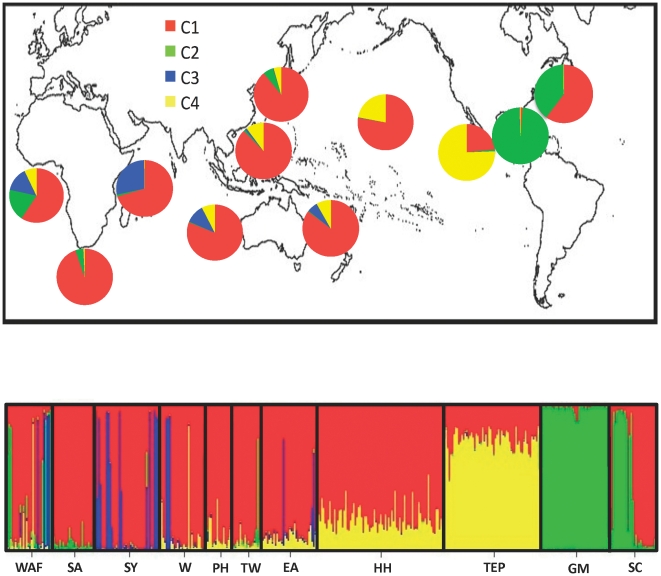
Proportions of population ancestry from each of four multi-locus lineages (C1–C4) defined by Structure [**52**]. Pie charts in the top figure indicate the relative proportion of individuals from each sampling site that assign to each lineage, and the bottom figure shows individual genotypic assignments in a conventional bar plot, organized by sampling site. TEP = Tropical East Pacific, HH = Hawaii, TW = Taiwan, PH = Philippines, EA = East Australia, W = West Australia, SY = Seychelles, SA = South Africa, WAF = West Africa, SC = South Carolina, and GM = Gulf of Mexico.

Structure [51] indicates four clusters or populations among our global data set (Figure 3). Following selection of *K*, we used Structure's individual genotypic assignments to generate pie charts for each sampling site, in addition to using the traditional bar plot to display individual admixture. The pie charts denote the total probability of individuals sampled from that site belonging to each of the four clusters indicated by Structure (C1–C4). Geographical structuring is as follows: Cluster C1 is by far the most widespread, represented in every sampling site from the TEP and Hawaii throughout the Indo-Pacific and Atlantic to South Carolina. Taiwan, Philippines, and South Africa assign primarily to this cluster, though this could be due to low sample size. One small, relatively isolated cluster (C3) is predominantly observed in the Indian Ocean, with high representation in the Seychelles. In South Carolina, about half of the individuals were assigned to C1, the widespread cluster found in the Indo-Pacific, and about half to the predominantly Atlantic C2 cluster shared between West Africa and the Gulf of Mexico. The low level of admixture in South Carolina indicates that samples may have been drawn from a common feeding area where distinct breeding populations may overlap. The Gulf collections were the most homogeneous of any sampling site, with nearly 100% of individuals assigned exclusively to C2. The TEP sample also exhibited high population homogeneity, with the majority of individuals assigned to a single genetic cluster (C4).

For the Analysis of Molecular Variance (AMOVA), we grouped sites into four subpopulations as indicated by Structure and tested these for within- and between-group variation. Individuals from the East Pacific and the Gulf of Mexico had the greatest probability of self-assignment and were accordingly analyzed as independent sub-populations. As the remaining nine sites showed extensive admixture, groupings were not immediately obvious and collection sites were therefore clustered by major biogeographic province, these being the Indo-Pacific (central Pacific – West Indian Ocean) and Atlantic [56]. The resulting AMOVA groupings were as follows: (i) Tropical East Pacific; (ii) Indo-Pacific (Hawaii, Taiwan, Philippines, East Australia, South Africa, Seychelles, and West Australia); (iv) Atlantic (South Carolina and West Africa); and (v) Gulf of Mexico. No significant variance was found among sampling sites within groups (*F*_SC_ = 0.002, *P* = 0.098). However, 4.88% of the variance was partitioned among groups (*F*_CT_ = 0.049, *P*<0.001), indicating that our groupings reflect actual population structure. Global genetic structure was low but significant (*F*_ST_ = 0.035, *P*<0.001). Though our data indicate gene flow both across the Sunda Shelf and around the tip of South Africa, the presence of a unique multi-locus lineage primarily found within the Indian Ocean (C3, blue in Fig. 3) indicates some isolation in this region, perhaps in the Gulf of Oman.

Genetic distances derived from biparentally-inherited microsatellite DNA (*F*′_ST_) and matrilineal mtDNA (mt*F*_ST_ and Φ_ST_) are contrasted in Table 4. Paired Student's t-tests revealed statistically significant differences between *F*′_ST_ and mt*F*_ST_ in *S. lewini* (t = −5.025, *P*<0.001). Φ_ST_ and mt*F*_ST_ were highly similar except in a limited number of pairwise comparisons where the magnitude of Φ_ST_ was elevated relative to mt*F*_ST_ (though *P* values remained consistent). This effect was noted particularly in pairwise comparisons that include the Seychelles, West Africa, and West Australia. Because divergent haplotypes coupled with genetic isolation could create a signal of haplotypic distance that increases the magnitude of Φ_ST_ relative to mt*F*_ST_, these data indicate some genetic isolation of Indian Ocean sampling sites relative to the rest of the globe, results similar to those indicated by Structure.

IMa revealed maximum likelihood estimates of the number of migrants per generation (Nm = θ*m*/4) between 0.03 and 7.22. Although 95% posterior probability densities (PPDs) of these estimates were large with several infinite upper boundaries, all PPDs had strong peaks with probabilities falling to zero as *m* approached zero. We report both Nm_1_ (estimated migrants per generation from population 2 into population 1) and Nm_2_ (estimated migrants per generation from population 1 into population 2) for each pair of sites examined, and compare these mixed-marker values to the migration rates for mitochondrial data alone (M) from Duncan et al. [13] (Table 4). Along continuous coastlines, Nm_1_/Nm_2_ were consistently equivalent to or lower than M, while across ocean basins Nm_1_/Nm_2_ values were higher than M, sometimes by an order of magnitude.

## Discussion

The distribution of maternal lineages indicates strong restrictions to dispersal between discontinuous coastlines, whereas biparentally-inherited markers reveal much higher connectivity and in some cases nonsignificant population structure across ocean basins. Male-mediated dispersal and gene flow has likely facilitated the contemporary connectivity observed among global *Sphyrna lewini* populations, while population genetic differences between sites are enhanced by females' ontogenetic requirement for coastal habitat. Populations at the ends of the range in the Tropical East Pacific (TEP) and the Gulf of Mexico are relatively young (<100,000 yr) [13], whereas the oldest contemporary populations, based on both mtDNA and microsatellite data, are in the central Indo-Pacific [13].

### Sex-biased dispersal

We identified marked differences between male and female dispersal patterns in *Sphyrna lewini*, as indicated by contrasting genetic distances derived from matrilineal mtDNA (mt*F*_ST_ and Φ_ST_; [13]) and biparentally-inherited microsatellite DNA (*F′*_ST_; Table 4). As males do not transmit mtDNA haplotypes to subsequent generations, high mtDNA structure and limited or absent microsatellite DNA structure between sites indicates female site fidelity, most likely for the purpose of reproduction.

Overall, *F′*_ST_ was marginally lower than mt*F*_ST_ and Φ_ST_ along continuous coastlines but an order of magnitude lower across ocean basins (Table 4). For example, highly significant mtDNA structure contrasts with a lack of significant structure in microsatellite DNA between the Seychelles in the western Indian Ocean and West Australia (*F′*_ST_ = −0.009, *P* = 0.149; mt*F*_ST_ = 0.521, *P*≤0.001; Φ_ST_ = 0.736, *P*≤0.001), and between Hawaii and all West Pacific sites (Table 4), indicating contemporary male-mediated gene flow across large expanses of open ocean. Male-biased dispersal was also noted across some biogeographic barriers, such as the Tropical East Pacific barrier (between Hawaii and the TEP) and Mid-Atlantic barrier (between East and West Atlantic). Notably, estimates of gene flow across the Atlantic (∼4000 km) were lower than across the Indo-Pacific (∼7000 km), as evidenced by significant genetic structure at microsatellite loci. There was on average an order of magnitude difference in population structure detected by mitochondrial and nuclear DNA in these pairs. Concordantly, IMa indicated that estimates of gene flow among mitochondrial and mixed-marker analysis are similar along continental margins, a finding consistent with generally high gene flow among both males and females along continuous coastlines. Across ocean basins, however, the estimates for mtDNA are far higher than for biparental markers (Table 4), indicating that when dispersal events across oceanic barriers occur, they are largely male-mediated.

Although divergent *F*_ST_ values gleaned from mitochondria are generally expected to be higher and easier to detect than nuclear *F*_ST_ because of mtDNA's haploid nature and uniparental inheritance [32], the analyses used in this study show marker divergences to be attributable to sex-biased dispersal rather than differences in N_e_. First, the large numbers of highly polymorphic markers used in this study lend considerable power to discerning genetic structure at nuclear loci, power that is equal to or greater than mtDNA. In their statistical comparisons between haploid and diploid marker types, Larsson *et al.* [32] demonstrated that in situations which include several populations, numerous loci, and many alleles per locus (>5), organelle and nuclear marker types have equal power to detect genetic structure. Second, tests for recent population expansion in Duncan et al. (2006) were consistently non-significant across all sampling sites with the exception of Hawaii. Evidence of stable populations across *S. lewini's* range decreases the likelihood that the order of magnitude or greater differences between mtDNA and nDNA structure, standardized for marker heterozygosity, are purely an artifact of differences in marker N_e_. Further, IMa estimates of gene flow, which are by design robust to differences in N_e_, show high migration at mixed markers compared with mtDNA values across ocean basins but not along continuous coastlines, results that are biologically consistent with our hypothesis of male-biased dispersal. Notably, both nuclear DNA markers and gene flow estimates from mixed-marker analysis (*Nm*) include evidence gleaned from biparental inheritance, yet even with 50% input from matrilines, the differences between marker types clearly signify male-mediated dispersal.

This male-biased dispersal pattern is consistent with the premise that shark reproductive strategies more closely resemble those of sea turtles and marine mammals than other fishes (Musick 1999). It is likely that female reliance on coastal habitat for reproduction can explain the relatively limited vagility of *S. lewini* females. One caveat to our conclusions is that genetic isolation is not always synonymous with restricted physical movement in sharks. Lemon sharks (*Negaprion brevirostris*) show philopatry to specific sites [25], [57], but may move extensively between breeding seasons. Similarly, contrasting mtDNA and microsatellite inheritance reveal male-biased dispersal between South Africa and Australia in white sharks (*C. carcharias*; [16]), however mature females have been shown to migrate between these locations [58]. In nature, males and females may have the same migratory circuit, but female migrations include reproductive philopatry whereas male migrations provide an opportunity for long-distance gene flow. Though mtDNA structure along continental margins is low when compared across ocean basins, both this work and others indicate female site fidelity to coastlines, and perhaps even philopatry to specific coastal embayments [13], [25], [59]. The annual reproductive cycle probably precludes long-distance movements by female *S. lewini*, but telemetry studies would be necessary to link the sex-biased gene flow observed here with reduced transoceanic movement by females.

### Global phylogeography

The statistical parsimony analysis in Duncan *et al.* [13] identified basal mtDNA haplotypes in the Indian and West Pacific Oceans, and mitochondrial coalescence analyses found the oldest populations of *Sphyrna lewini* in the same region (Table 1 and Figure 2 in [13]). These data further demonstrate that the East Pacific and Gulf of Mexico/West Atlantic sites have high pairwise structure and the lowest population ages, indicating relatively recent origins of contemporary populations [13]. Our dendrogram of microsatellite *F*_ST_ distances (Figure 2) shows that Indo-Pacific sites group together with high allelic similarity, with differential branching into the Tropical East Pacific and Atlantic Oceans. We conclude that the Indo-Pacific, and possibly the West Pacific, is the origin of modern *S. lewini*, with subsequent divergence eastward into the East Pacific and westward into the Atlantic. These findings are consistent with the IWP Center of Origin hypothesis proposed by Briggs [60] and advocated by Duncan *et al.* [13]. Notably, microsatellite allelic richness and admixture is highest within the Indo-Pacific (*A_r_* = 7.30−8.49), and decreases progressively with distance from that region (R-square of *A_r_* = 0.43, *P* = 0.028). Genetic structure shows the opposite pattern, with lower population structure within the Indo-Pacific and increasing differentiation towards the ends of the global range in the East Pacific and Gulf of Mexico. These sites each show the lowest heterozygosity, admixture, and allelic richness, as well as the highest pairwise differentiation (*F′*_ST_ = 0.519, *P*<0.001).

Despite evidence of male migration across thousands of kilometers of open ocean, we identified surprisingly strong genetic differentiation between the Gulf of Mexico and the adjacent South Carolina (West Atlantic) population. Structure shows almost zero admixture in the Gulf population, while the adjacent South Carolina (West Atlantic) site shows a near 1∶1 mix of Gulf (green) and Indo-Pacific (red) types (Figure 3). IMa indicates unidirectional dispersal between these two sites, with estimated migration out of the Gulf of Mexico into the West Atlantic being high relative to other sites (Nm = 7.22), while estimated migration into the Gulf is near zero (Nm = 0.26; Table 4). This pattern is concordant among marker types, indicating restricted gene flow into the Gulf of Mexico for both males and females. However, a similar study on *S. lewini* mtDNA using samples from adult sharks taken in the shark fin trade [59] found no such structure separating the Gulf from the West Atlantic. The source of this discrepancy may lie in the fact that Chapman et al. [59] sampled adults, possibly from a mixed pool of transient migrants. In addition to adults, the current study includes unrelated neonates and juveniles, which are less dispersive and therefore more likely to reflect to the genetic consequences of philopatry.

Reasons for the isolation of the Gulf of Mexico from the proximate West Atlantic in the current study are hard to pinpoint, though it is clear that these samples belong to *S. lewini* and not the cryptic Sphyrnid species found in the same region [61]. Duncan *et al.* [13] proposed that partitioning throughout the West Atlantic, Gulf of Mexico, and Caribbean was largely driven by the distances between nursery sites. Long-distance tagging data on hammerhead sharks are few, and only one *S. lewini* tagged in the Gulf of Mexico (sex unknown) has been recaptured to date, not far from the catch site [18], [23]. Similar to the Atlantic, low but significant structure was found between West and South Africa, and between the Philippines, Taiwan, and East Australia (Table 4), possibly reflecting the input of strong female philopatry.

### Biogeographic barriers

The circumglobal tropical distribution of *S. lewini* crosses multiple well-documented marine biogeographic barriers and includes seamounts, coralline archipelagos, pelagic blue-water environments, and coastal brackish-water estuaries. In *S. lewini*, maximum population differentiation between East Pacific and West Atlantic populations is likely due to the difficulty of maintaining circumglobal gene flow, in particular the bridging of large stretches of open water separating the Tropical East Pacific from the Central Pacific, the cold upwelling water around South Africa, and the oceanic gap between East and West Atlantic [62]. Two biogeographic barriers require particular attention here: the Indo-Pacific Barrier and the Isthmus of Panama.

The shallow continental shelf between the Pacific and Indian Oceans is regarded as a substantial barrier to tropical invertebrates and fishes [63], [64], [65], [66]. At glacial maxima, the Sunda Shelf between southeast Asia and Australia/New Guinea is exposed and forms a nearly impenetrable land bridge between Pacific and Indian Oceans. However, the transient nature of this Indo-Pacific Barrier produces different responses by organisms with a variety of dispersal mechanisms. Gaither *et al.* [67] noted that most teleost (bony) fishes surveyed across this barrier showed strong population-level or evolutionary separations. However, this does not seem to be the case for the elasmobranchs surveyed to date (Keeney & Heist 2006; Castro *et al.* 2007), possibly because their high adult vagility allows for connectivity across the Sunda Shelf, even during lowered sea levels. While the scalloped hammerhead shows a population level separation across this barrier with mtDNA, that lack of microsatellite DNA structure over the same area demonstrates male mediated dispersal between the Pacific and Indian Oceans (Table 4).

The Isthmus of Panama separated the East Pacific from the West Atlantic approximately three million years ago [68], [69]. Mitochondrial coalescence estimates for the TEP and Gulf of Mexico populations show probable range expansion into both areas long after the rise of the Isthmus [13], indicating that these highly differentiated populations have never been in contact and are linked only through circumglobal gene flow across three ocean basins. In this case, the Isthmus differs from its well-documented role as an emergent vicariant barrier isolating portions of a formerly contiguous population [68]. Instead, we find that *S. lewini* is prevented from completing its circumglobal range expansion by secondary vicariance due to the Isthmian land barrier. In *S. lewini*, the secondary vicariant role of the Isthmus of Panama represents a novel and previously undescribed role for the Isthmus.

Notably, such a vicariant speciation event has already marked the evolutionary history of ancestral *S. lewini*; despite an Indo-Pacific origin, a cryptic sister species was recently discovered in the West Atlantic [61], a likely product of an earlier Atlantic colonization. The West Atlantic and TEP are also sites of endemism for several coastal members of the genus *Sphyrna* [22], indicating that a shared biogeographic mechanism, potentially linked to the presence of the Isthmus of Panama, is driving Sphyrnid diversity in this region.

### Conclusions and management implications

The scalloped hammerhead shark (*Sphyrna lewini*) is a large, mobile predator with a coastal-pelagic life history and global range. Both mtDNA and microsatellite data indicate that modern populations of this tropical species originated in the Indo-West Pacific, then subsequently dispersed into the Atlantic and East Pacific. Although the ability to traverse pelagic habitat indicates high rates of dispersal, female *S. lewini* display site fidelity to single coastlines, archipelagos, or individual nursery areas and show no evidence of ongoing trans-oceanic movement. Male *S. lewini*, by contrast, disperse long distances across the open ocean with clear consequences in reproduction and the transmission of gametes, though the frequency of these migrations is unknown. Populations at the ends of the range in the TEP and the Gulf of Mexico have differentiated over time, and are prevented from contact by the Isthmus of Panama. Currently scalloped hammerheads are exposed to massive fishery mortality on a global scale, and their coastal-pelagic life history makes them vulnerable to inshore and estuarine fishing mortality as well as offshore commercial operations. In 2008, the IUCN raised the global status of *S. lewini* from “threatened” to “endangered” [24]. In the U.S., scalloped hammerheads are grouped with the large coastal species (LCS), the category of sharks that scientists consider most susceptible to commercial overfishing [70]. This group has undergone documented declines throughout the Atlantic, where *S. lewini* is considered to be a single genetic stock [24], [71], a premise that is clearly refuted by population genetic data presented here and in previous work [59].

In regards to management strategies, the complex population structure observed in *S. lewini* highlights the need for analyses of genetic markers with multiple lines of inheritance. Single-marker assays using either female or biparentally-inherited loci alone conducted on species with complex reproductive behavior and life history may give a misleading picture of management units [12], where multiple-locus studies offer more comprehensive results. While mtDNA data from philopatric females may reflect genetic partitioning within ocean basins or along continental margins, arguing for more conservative management units along coastlines, highly dispersive males may be crossing oceans and are potentially being fished far from their location of origin and at both ends of a single migratory circuit. Overall, our evidence of differential dispersal patterns in male and female *S. lewini*, as well as strong genetic partitioning in the West Atlantic and in other regions of the world, leads us to recommend drastic reevaluation of management units and conservation strategies for this species.
